# A Third Generation Calphad Description of Pure Lithium

**DOI:** 10.3390/ma17194750

**Published:** 2024-09-27

**Authors:** Wenjun Xu, Xiaobo Li, Mingyu Ou, Jinning Ma

**Affiliations:** School of Materials Science and Engineering, Xiangtan University, Xiangtan 411105, China; 202221551543@smail.xtu.edu.cn (W.X.); 202221551607@smail.xtu.edu.cn (M.O.); 202221551606@smail.xtu.edu.cn (J.M.)

**Keywords:** lithium, extend Einstein model, extend Debye model, SGTE database, heat capacity

## Abstract

This study, based on the analysis of existing experimental data, presents a third generation Calphad description of lithium, covering all temperature ranges, using nonlinear least squares in Matlab. We have expanded the SGTE database’s description of lithium phases (face-centered cube, body-centered cube, liquid) down to 0 K with reasonable accuracy, taking into account the significant effort required to reconstruct the database for each element. During the evaluation process, it was determined that the low-temperature phase of lithium is fcc. The heat capacity of crystalline Li was accurately described using the extended Debye model. The third generation Calphad description of lithium utilized the two-state model and the extended Einstein model, leading to improved agreement with experimental data compared to previous assessments.

## 1. Introduction

The SGTE (Scientific Group Thermodata Europe) pure element database has been widely adopted as the basis for multi-component thermodynamic models by international associations [1]. Currently, most thermodynamic data for pure elements in the temperature range of 0 to 298.15 K has not been evaluated in this database. To address this issue, a physically meaningful model was first proposed at the Ringberg workshop in 1995 to describe solid and liquid phases [2]. Chen and Sundman [3] have described pure Fe based on this model. Lithium is extensively utilized in numerous fields and constitutes the primary element of lightweight alloys. Moreover, if it is to be employed in harsh environments, such as outer space, a thermodynamic description within the range of 0 to 298.15 K needs to be provided. Nevertheless, the thermodynamic characteristics of the low-temperature stable and metastable phases of lithium have not been comprehensively assessed.

In recent years, numerous researchers have introduced various methods for developing third-generation Calphad databases. Roslyakova et al. [4] accurately evaluated the thermodynamic properties of pure Cr, Fe, and Al using a segmented regression (SR) model. Yuxun Jiang et al. [5,6] utilized a physically based segmented regression model and considered the thermodynamic properties of all experimental phases of the Cr-Nb and Cr-Ta systems to optimally evaluate and extend these systems to 0 K. EnKuan Zhang [7] et al. also employed this model to conduct a third-generation thermodynamic description of the Mo-Nb-Ta-W system. A. Obaied et al. [8] re-evaluated the unary description of Cr using a modified segmented regression model (MSR). In the study by Voronin and Kutsenok [9], multiple Einstein functions were initially proposed to fit heat capacity, with the extended Einstein and two-state models also used for the third-generation thermodynamic description. For instance, Khvan [10,11,12] accurately evaluated the thermodynamic properties of pure Au, Pb, and Cu, and Zhangting He [13,14,15] assessed the phases of W (bcc, fcc, hcp, and liquid) as well as the W-C and Al-C binary systems.

Currently, there are two primary approaches for third-generation Calphad descriptions. One approach involves using physically meaningful models for a comprehensive description, while the other approach involves extending the existing SGTE database in a reasonable manner [16]. In this study, we utilize the extended Einstein model and the two-state model to comprehensively describe lithium. Additionally, we employ the extended Debye model to accurately depict the heat capacity of the solid phase, and apply the extended Einstein model to reasonably expand the lithium phase (fcc, bcc, liquid) in the SGTE database down to 0 K.

## 2. Literature Review

### 2.1. Low-Temperature Phase Transition of Li

One of the challenges in describing the heat capacity of lithium lies in the low-temperature phase transition it undergoes. There has been a longstanding debate over which structure, 9R or fcc, represents the stable low-temperature phase. The transition from bcc to fcc at low temperatures was initially observed by Barrett [17] through X-ray diffraction, revealing that this transition occurs when lithium metal undergoes plastic deformation at around −196 °C. Subsequent studies [18] uncovered a hexagonal close-packed arrangement as the phase below 70 K. The 9R structure as a low-temperature phase of lithium was suggested by Overhauser et al. [19]. Harris et al. [20] indicated that the 9R to bcc structure in lithium by spin relaxation spectroscopy study of mu meson is approximately 73 K. In 1997, Kara et al. [21] analyzed neutron scattering data and concluded that below 80 K, the disordered polymorphic structure of lithium is composed of short-range correlated fcc and hcp phases, coexisting with the longer-range ordered 9R structure. Their results indicate that fcc is the most stable phase at low temperatures. Additionally, according to the research of Schwarz and Blaschko [22], the formation of the 9R structure is most likely a consequence of the alleviation of macroscopic shear stresses, which leads to the nucleation of the fcc structure in the bcc crystal. Therefore, the 9R structure can be regarded as an fcc lattice with stacking faults after each ABC layer, significantly reducing the macroscopic shear stresses in the original fcc lattice.

Pichl et al. [23] studied the low-temperature martensitic transformation of lithium and found that below the martensitic temperature, a long-range ordered 9R structure, a one-dimensional disordered polymorphic phase, and an intermediate phase fcc in a hysteresis state appear upon heating. Through their research, they concluded that the fcc structure is the true equilibrium phase, while the 9R structure is a metastable phase. Ackland et al. [24] used a multiscale simulation approach combining synchrotron X-ray diffraction and density functional theory with molecular dynamics to demonstrate that the widely recognized 9R phase is in fact a metastable state and that the ground state is the fcc phase.

### 2.2. Heat Capacity of the Crystalline and Liquid Phases of Li

**Figure 1:**
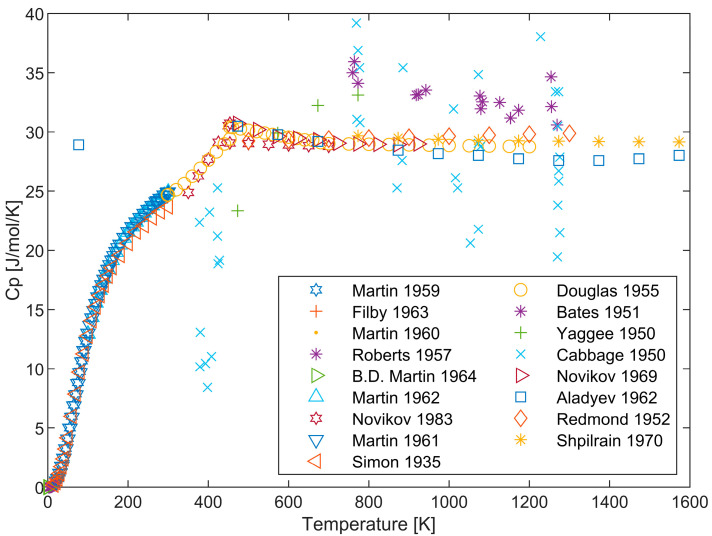
Experimental heat capacity data for Li [26,34,35,36,37,38,39,40,41,42,43,44,45,46,47,48,49].

The thermodynamic properties of pure lithium have been reviewed multiple times [25,26,27,28,29,30,31,32,33], with the heat capacities measured by Martin [34] and Douglas [35] being widely accepted as the main data sources. Alcock et al. [33] conducted the most comprehensive work to date, summarizing extensive experimental data on Li conducting comparative analysis. The recommended heat capacity data and enthalpy data are essentially consistent among these reviews. Figure 1 presents the experimental value of the heat capacity of lithium. The sources of experimental heat capacity data used in the present study, along with their respective relative uncertainties, sample purities, and experimental methods, are all listed in Table 1. The comparison between our evaluation results and those of others is presented in Table 2.

#### 2.2.1. Low Temperature Data

The primary reference data for low temperatures are the heat capacity of a 99.95% pure lithium sample, measured using adiabatic calorimetry in the temperature range of 20–300 K by Martin et al. [34,36], which is consistent with experimental results obtained by Simon and Swain [38]. Furthermore, Martin et al. [37] claimed to have further improved the automatic calorimeter, originally proposed by Dauphinee et al. [51] for continuous heating measurements, by allowing the use of platinum resistance thermometry, resulting in a measurement accuracy of 0.1%. They measured the heat capacity of lithium in the temperature range of 100–300 K. Data near 0 K are provided by low-temperature measurements of electron heat capacity [39,40,41,42]. The data measured in studies [40,41,42] exhibit high consistency. Martin et al. [39] reported a lower value for the heat capacity compared to theirs, which is likely to be inaccurate due to the low Debye temperature selected.

#### 2.2.2. Bcc and Liquid Phase

The low-temperature phase transition of lithium occurs approximately around 80 K, and the heat capacity data between 80 K and 453.69 K are provided by the references [34,35,36,37,38,46,51]. Liquid phase heat capacity data are provided by [26,35,43,44,45,46,47,48,49] and the discrepancy of the measured heat capacity in [46,47,48,49] is within 7%, where the dispersion of individual data points are quite large. Cabbage [45] conducted an enthalpy measurement of liquid Li in a drop calorimeter, ranging from 780 to 1280 K. The experiment utilized a commercial 99% Li material from Eimer and Amend. Prior to and after the experiment, samples were analyzed and found to contain up to 0.37 wt.% Li_2_O and 0.92 wt.% LiN, which caused the obtained results to deviate from the normal trend and there was a large error. Douglas et al. [51] utilized an ice calorimeter and drop method to determine the heat capacity of 99.9% pure lithium within the temperature range of 298.16–1200 K. From a comprehensive perspective on materials, experimental instruments, and methods, the experimental data measured by [51] appears to be the most detailed and accurate. Therefore, this paper selects the experimental data as the primary data set for fitting the liquid phase.

### 2.3. Enthalpy Data

**Figure 2:**
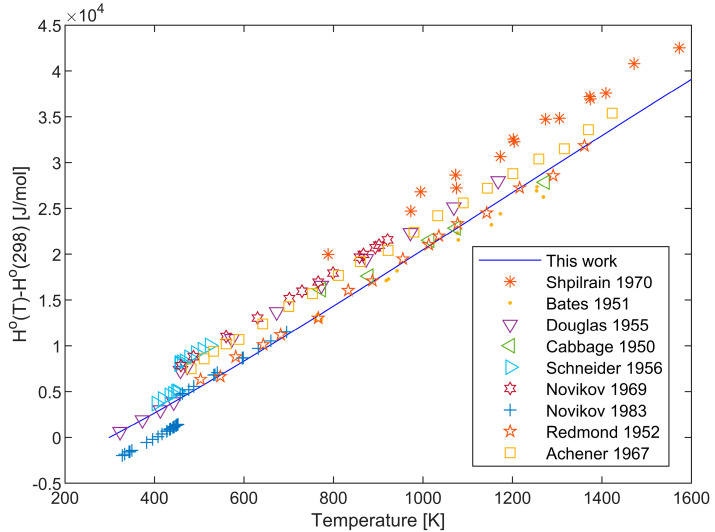
Enthalpy increment measurements for Li H^o^(T)-H^o^(298.15) and their comparison (Values were recalculated from original temperatures to 298.15) [26,35,43,45,46,47,49,52,53].

The enthalpy values of the lithium crystal phase and liquid phase have been comprehensively reviewed in [33], with a detailed comparison of the relevant data. The enthalpy data of [26,35,47,49,52,53] exhibit good consistency, while the enthalpy measured by [43,45] deviates from the general trend, showing large errors at individual data points. Figure 2 shows the enthalpy increment calculated by the extended Einstein model compared with the experimental data.

## 3. Thermodynamic Model

### 3.1. Extended Debye Model and Extended Einstein Model

The pure element Gibbs energy can be derived from its heat capacity, and this modeling method has become the main tool for developing physically-based thermodynamic property models in the third-generation Calphad database. We know that polynomial models cannot well describe the heat capacity from 0 to 298.15 K. In order to obtain physically meaningful results, the following methods can be used: the isochoric heat capacity of crystal vibrations can be described using the Debye model or the Einstein model. By correcting from isochoric heat capacity to isobaric heat capacity and adding the contribution of electronic heat capacity, for non-magnetic pure metals (with zero magnetic heat capacity), their isobaric heat capacity can be expressed using Equation (1) as follows:

(1) $$C_{p}=C_{p}^{electron}+C_{p}^{lattice}=\gamma T+C_{V}^{lattice}+TB_{T}V\beta^{2}=\gamma T+C_{v}^{Ein}+TB_{T}V\beta^{2}=\gamma T+C_{v}^{Deb}+TB_{T}V\beta^{2}$$

Here, $C_{p}^{electron}$, $C_{p}^{lattice}$, and $C_{v}^{lattice}$ represent the electronic isobaric heat capacity, lattice isobaric heat capacity, and lattice isochoric heat capacity, respectively. $C_{v}^{lattice}$. $C_{p}^{Deb}$, and $C_{p}^{Ein}$ are heat capacities at constant volume described by the Debye model (2) and the Einstein model (3), respectively. *γ* represents the electronic heat capacity coefficient, *R* is the gas constant, *B_T_* is the isothermal bulk modulus, *V* is the molar volume, and *β* is the coefficient of volumetric thermal expansion.

(2) $$C_{v}^{Deb}\left(T,\Theta_{D}\right)=9R\left({\frac{T}{\Theta_{D}}}^{3}\right)\int_{0}^{\Theta_{D}/T}\frac{x^{4}e^{x}}{{\left(e^{x}-1\right)}^{2}}dx$$

(3) $$C_{v}^{Ein}\left(T,\Theta_{E}\right)=3R\left({\frac{T}{\Theta_{E}}}^{2}\right)\frac{e^{\Theta_{E}/T}}{{\left(e^{\Theta_{E}/T}-1\right)}^{2}}$$

*Θ_D_* and *Θ_E_* are the Debye temperature and Einstein temperature, respectively. Although the best-available physical models are used to describe heat capacity, it is difficult to obtain the necessary physical property values under existing experimental conditions. Therefore, the model for describing the heat capacity of metallic lithium is simplified as follows:

(4) $$C_{p}=C_{v}^{Deb}(T,\Theta_{D})\;\mathrm{or}\;C_{v}^{Ein}(T,\Theta_{E})+aT+bT^{n}+cT^{m}$$

Here, the second term includes contributions from electronic excitations, low-order anharmonic lattice vibrations, and a *C_P_* to *C_V_* correction related to volume differences. The third term is contributed by high-order anharmonic lattice vibrations, where the value of *n* can be chosen from 2, 3, and 4. The fourth term is introduced to enhance the fit to the heat capacity curve. In this study, the extended Debye model and extended Einstein model have *n* and *m* values of 2 and 4, and 2 and 3, respectively. The parameters *a*, *b*, *c*, Debye temperature, and Einstein temperature are determined by fitting experimental data, with a constraint that the value of a approximates the electronic heat capacity coefficient of lithium, and the Debye and Einstein temperatures do not exceed their respective limits.

Thermodynamic quantities that can be calculated from heat capacity include

(5) $$\begin{array}{l} H=\int_{p}^{T}C_{p}+H(0K)=\int_{0}^{T}C_{p}dT-\int_{0}^{298.15}C_{p}dT+H(298.15K)\\ S=\int_{0}^{T}\frac{C_{p}}{T}dT+S(0K)\\ G=H-TS \end{array}$$

As Debye model does not have an analytic formula, here we only provide the *H_Ein_*, *S_Ein_*, and *G_Ein_* values obtained from the extended Einstein model:

(6) $$H_{Ein}=3/2R\Theta_{E}\frac{\exp(\Theta_{E}/T)+1}{\exp(\Theta_{E}/T)-1}+\frac{a}{2}T^{2}+\frac{b}{3}T^{3}+\frac{c}{4}T^{4}-H_{ref}+E_{0}$$

(7) $$S_{Ein}=3R\frac{\Theta_{E}}{T}\frac{\exp(\Theta_{E}/T)}{\exp(\Theta_{E}/T)-1}-3R\ln(\exp(\Theta_{E}/T)-1)+aT+\frac{b}{2}T^{2}+\frac{c}{3}T^{3}$$

(8) $$G_{Ein}=\frac{3}{2}R\Theta_{E}-3RT\ln\frac{\exp(\Theta_{E}/T)}{\exp(\Theta_{E}/T)-1}-\frac{a}{2}T^{2}-\frac{b}{6}T^{3}-\frac{c}{12}T^{4}-H_{ref}+E_{0}$$

Here, *H_ref_* represents the enthalpy value at 298.15 K, and *E*_0_ is optimized throughout the fitting process.

### 3.2. Two-State Model

The liquid phase is represented by a two-state thermodynamic model [54,55] to describe its thermodynamic properties. According to this model, the liquid phase is assumed to be composed of two types of ideal atoms in different states: the translational state atoms in the liquid-like phase and the vibrational state atoms in the solid-like phase. The solid-like atoms can be considered as representatives of pure amorphous phases. This two-state model describes the entire temperature range of liquid amorphous phases well. At a certain temperature, these two types of atoms can reach equilibrium. Let *ε* denote the number of liquid-like phase atoms, and 1-*ε* denote the number of solid-like phase atoms. When the temperature changes, the two states of atoms will transform into each other, thus achieving equilibrium between the two parts of atoms. When the equilibrium is reached, *∂G_L_*/*∂ε* = 0, the value of ε is determined by the following equation:

(9) $$\varepsilon =\frac{e^{-\Delta G_{dif}/RT}}{1+e^{-\Delta G_{dif}/RT}}$$

where the ∆*G_dif_* represents the difference in Gibbs energy between the atomic species in the liquid-like and solid-like states. Ågren [56] applied a bimodal approach to describe the thermodynamic properties of liquid Sn, and found that a more complex temperature-dependent model was required to accurately describe the formation of defects for a better fit. They provided a general expression for which ∆*G_dif_* can be expressed as

(10) $$\Delta G_{dif}=G^{liq}-G^{sol}=A-BT+CT\ln T\ldots$$

The Gibbs free energy of the solid phase is determined through an approximation of its heat capacity, which is described by the Einstein or Debye models with additional items as mentioned earlier. Here, we use the extended Einstein model to calculate it.

(11) $$C_{p}^{sol}=3R{\left(\frac{\Theta_{E}}{T}\right)}^{2}\frac{e^{\Theta_{E}/T}}{{\left(e^{\Theta_{E}/T}-1\right)}^{2}}+aT+bT^{2}+cT^{3}$$

The initial value of *Θ_E_* matches the Einstein temperature of solid lithium, while the parameters *a*, *b*, and *c* are determined by fitting the entire liquid phase. The Gibbs free energy of the entire liquid phase can be expressed by the following equation:

(12) $$G^{L}=G^{sol}+\varepsilon \Delta G_{dif}+RT\left[\left(1-\varepsilon \right)\ln\left(1-\varepsilon \right)+\varepsilon \ln\varepsilon \right]$$

Substituting *ε* into the Equation (12) gives

(13) $$G^{L}=G^{sol}-RT\ln(1+\exp(-\frac{\Delta G_{dif}}{RT}))$$

From this (13), the liquid phase entropy and heat capacity can be derived:

(14) $$S^{L}=S^{sol}+(\frac{A}{T}-C)\varepsilon +R\ln(1+\exp(-\frac{\Delta G_{dif}}{RT}))$$

(15) $$C_{p}^{L}=C_{p}^{sol}+\left(A-CT\right)\frac{d\varepsilon }{dT}-C\varepsilon$$

## 4. Results

### 4.1. Fitting by the Extended Debye Model

**Figure 3:**
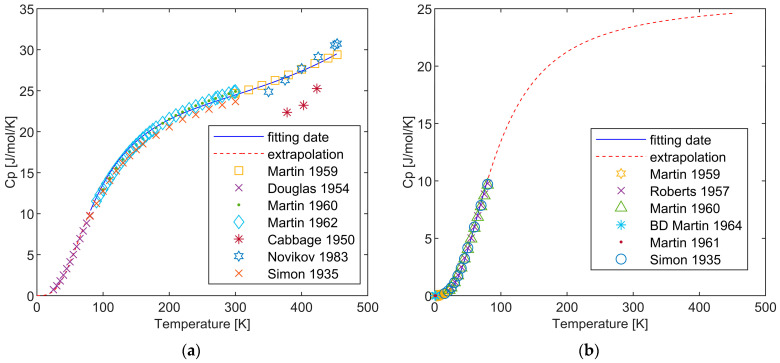
Heat capacity of fcc (**a**) and bcc (**b**) obtained from the extended Debye model and their comparison with the experimental data [34,35,36,37,38,39,40,41,45,47].

In the Section 2, a review was conducted on the low-temperature phase transition of Li, determining fcc as the low-temperature phase, and fitting both the low-temperature and high-temperature phases using the extended Debye model. The temperature range for Li’s low-temperature phase (fcc) was selected from 0 K to 80 K, and the heat capacity of this phase was fitted accordingly. The bcc phase was fitted and extrapolated within the range of 80 K to 453.69 K. The calculated results for both phases, and their comparison with experimental data, are presented in Figure 3. The determination of the *Θ_D_* value is carried out during the fitting process by initially assuming a value (typically below the limiting Debye temperature for lithium), comparing the heat capacity curve obtained with the experimental data, and ensuring that the Gibbs free energy curve derived from the heat capacity satisfies the phase transition relationship, thereby obtaining the most optimal temperature value for the fit. Detailed parameter data can be found in Table 3. As the Debye model is unable to provide an analytical expression for the Gibbs free energy, only the expression for the heat capacity can be offered here. The fitting results for both phases align perfectly with the experimental data.

### 4.2. Overall Description by Extended Einstein Model and Two-State Model

**Figure 4:**
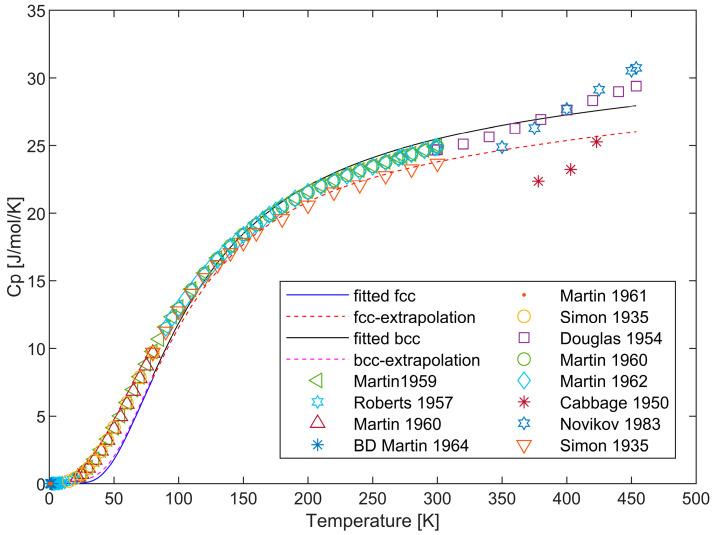
Heat capacity of crystal phases and their comparison with the experimental data [34,35,36,37,38,39,40,41,45,47].

**Figure 5:**
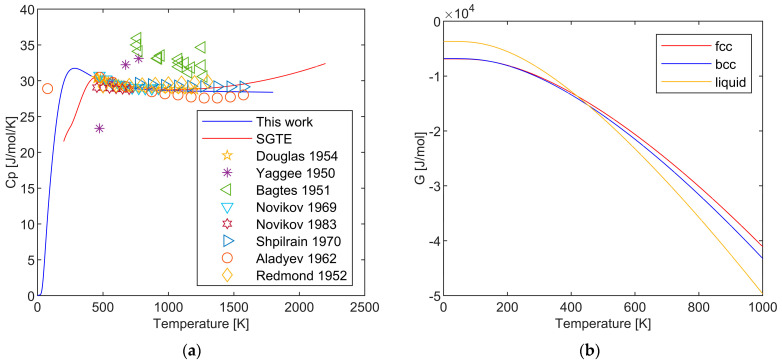
(**a**) The heat capacity of the liquid phase obtained by the two-state model and its comparison with experimental values and SGTE; (**b**) the Gibbs free energy of Li obtained from the overall description [26,35,43,44,46,47,48,49].

Due to the inherent limitations of the Debye model, which hinder further applications, an extended Einstein model is employed in fitting the heat capacities of fcc and bcc phases in the overall description. Consistency with the extended Debye model is maintained in the data utilized. Additionally, a dual-state model is applied for the liquid phase of lithium. The fitting results of the heat capacities for the solid phases are presented in Figure 4, while the heat capacity of the liquid phase obtained by the two-state model and the Gibbs free energy are illustrated in Figure 5. The derived parameters are detailed in Table 4. It is evident that, in comparison to the results from the SGTE, the use of the dual-state model provides a more favorable description of the liquid phase of Li and better aligns with the observed variations in experimental heat capacity. Meanwhile, the low-temperature heat capacity of the solid phase is less effectively described than that by the extended Debye model.

### 4.3. Extension of the SGTE Database

Extrapolation of the bcc, fcc, and liquid phases of pure lithium in the current SGTE database is conducted in a reasoned manner. In order to ensure a smooth transition at the connection point (*T* = 200 K) between the evaluated model and the high-temperature SGTE pure metal database, certain constraints have been introduced as follows:

(16) $$\begin{array}{l} C_{p}(fit)=C_{p}(SGTE)\\ S(fit)=S(SGTE)\\ dC_{p}(fit)/dT=dC_{p}(SGTE)/dT \end{array}$$

**Figure 6:**
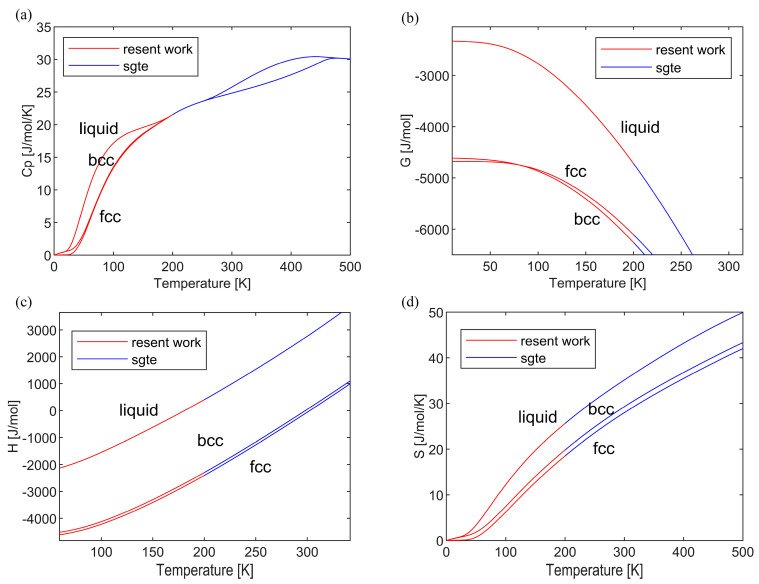
The temperature dependence of Gibbs energy (**a**), heat capacity (**b**), enthalpy (**c**), and entropy (**d**) of lithium phases (fcc, bcc, liquid) for extended SGTE.

Furthermore, it is aimed at aligning the enthalpy (at 298.15 K as the reference state) at the connection point with that of the SGTE pure metal database, where *dC_p_*/*dT* denotes the slope of heat capacity. Meanwhile, ensure that the Gibbs free energy curve derived from heat capacity adheres to the phase transition relationships of each phase. The interface conditions of the post-fitting *C_p_*, *G*, *H*, and *S* are depicted in Figure 6, with the detailed parameters presented in Table 5.

## 5. Discussion

It is important to note that this study utilized the nonlinear least squares method in MATLAB (R2021a) for fitting. The thermodynamic properties of lithium crystal phases have been evaluated using the extended Debye and extended Einstein models, both of which have their own advantages and disadvantages. Both models require relatively few parameters and show good consistency with the experimental data. The determination of the n and m values in models are based on the degree of agreement between the fitting curve and the experimental data. Additionally, it is necessary to comprehensively consider the use of several terms and their respective powers for different elements. The parameters in both models are determined through fitting, with the extended Debye model being more accurate. The extended Debye model can provide electron heat capacity coefficients and average Debye temperatures, but it is computationally complex and requires approximation using power series at low temperatures, with low-temperature thermodynamic data only represented in terms of heat capacity. In contrast, the evaluations from the extended Einstein model are less accurate between 0 and 100 K. However, the analytical expression of the Gibbs free energy in this model can be utilized for the optimization assessment of multicomponent phase diagrams, extending down to 0 K.

## 6. Conclusions

The crystal phase of lithium is described by the extended Debye model. The third-generation thermodynamic description of lithium utilized the two-state model and the extended Einstein model, leading to improved agreement with the experimental data compared to previous assessments, and can be utilized for the optimization assessment of multi-component phase diagrams involving lithium, extending down to 0 K. A reasonable extrapolation of the phases of lithium in the SGTE database (fcc, bcc, liquid) has been carried out, ensuring a smooth transition at the junction points.

## Figures and Tables

**Table 1:** Experimental determination of heat capacity.

Temperature Range, K	Relative Uncertainty, %	Purity as Reported in the Original Publications, %	Method	Ref.
20–300	0.2 above 80 K, 2% below 20 K	99.93 ^6^Li, 99.99 ^7^Li	Adiabatic calorimetry	D. L. Martin [36] 1959
20–300	0.2 above 80 K, 2% below 20 K	^b^ Natural Li (99.95%)	Adiabatic calorimetry	D. L. Martin [34] 1960
100–300	Reported to be 0.1	^b^ Natural Li (99.95%)	Continuous-heating method of calorimetry	D. L. Martin [37] 1962
15–300	^a^ 4	n/a	Calorimetry method	Simon [38]
0.4–1.5	Reported to be 5	^b^ Natural Li (99.95% and 99.3%)	Adiabatic calorimetry	D. L. Martin [39] 1961
0.35–2	n/a	99.999	Low-temperature calorimetry	B. D. Martin [40]
1.5–20	Reported to be 2	99. 5	Calorimetry method	Roberts [41]
3–30	Reported to be 0. 5	Natural Li ^b^ (99.95%)	Adiabatic calorimetry	Filby [42]
298.16–1200	Reported to be 5	99.9 0. 028%O, 0.003%N, 0.0036%Fe, 0.0006%Ni, 0. 029%Ca, 0.016%Na	Bunsen ice calorimetry and a drop method	Douglas [35]
760–1269	Reported to be 5	98.5	Bunsen ice calorimetry	Bates [43]
473–733	Reported to be 10	A commercial grade manufactured by the Maywood Chemical Company, Maywood, NJ, USA	Comparing the cooling rates in air of thin-wall stainless steel capsules	Yaggee [44]
378–1276 773–1273	n/a	99 0.37% Li_2_O, 0.92% LiN	The drop method	Cabbage [45]
473–923	Reported to be 0.3	99.33 0.38% Na, 0.14% Mg, 0.01% K and Al, 0.001% Fe, 0.003% Ca, 0.005% heavy metals, 0.012%N	Ice drop calorimetry	Novikov [46] 1969
350–750	0.6	99.5 0.06%Na, 0.005%K, 0.02%Mg, 0.03%Ca, 0.001%Mn, 0.005%Fe, 0.003%Al, 0.01%S_i_O_2_, 0.05%N (nitrides)	The drop method	Novikov [47] 1983
473–1573	Reported to be 8	99.5 0.5% Na and 0.02% K	Isothermal calorimetry	Aladyev [48]
500–1300	Reported to be 5	n/a	Bunsen ice calorimetry	Redmond [49]
773–1573	Reported to be 2	99 0.26%Na, 0.001%K, 0.003%Ca, 0.0072 N, other impurities < 0.015%	Boiling-point calorimetry	Shpil’rain [26]

^6^Li: main impurities 0.01%Mg, 0.01%Ba, 0.01%Sr, 0.02%Na, 0.02% Cu, 0.05%Fe, 0.025%Ca. ^7^Li: main impurities (Na, K, Rb, Cs) less than 0.001%. ^a^ The instrument used is generally consistent with that of Cristescu and Simon [50]. ^b^ main impurities K 0. 01%, Ca 0.01%, Na 0.005%, Fe 0.001%.n/a: no information in the original paper.

**Table 2:** Comparison of standard thermodynamic functions for lithium with suggested values.

Ref.	*C_p_*(298.15) [J/mol/K]	S^o^(298.15) [J/mol/K]	H^o^(298.15)-H^o^(0) [J/mol]	∆_fus_H [J/mol]	Annotation
This work	25.44	27.46	4518.8	3003	Extended Debye model
	24.44	28.81	4568.9	-	Extended Einstein model
SGTE [1]	24.79	29.12	4632	2999.93	
Hultgren [28]	26.148	29.275	1106 (cal/mol)	710 ± 10 (cal/mol)	
Chase [29]	24.623	29.085	-	3000 ± 15	
Alcock [33]	24.78 ± 0.1	28.99 ± 0.3	4671 ± 30	3000 ± 30	

**Table 3:** Assessed parameters and description of heat capacity obtained by Extended Debye Model.

Phase	Expression of *C_p_*	*a*	*b*	*c*	*Θ_D_*
fcc	$\begin{array}{l} C_{p}=9R{\left(\frac{T}{380}\right)}^{3}\int_{0}^{\frac{380}{T}}\frac{e^{x}x^{4}}{{\left(e^{x}-1\right)}^{2}}dx+\\ 0.0016T-9.9996\times 10^{-7}T^{2}\\ +2.2225\times 10^{-14}T^{4} \end{array}$	0.0016	−9.9996 × 10^−7^	2.2225 × 10^−14^	380
bcc	$\begin{array}{l} C_{p}=9R{\left(\frac{T}{375}\right)}^{3}\int_{0}^{\frac{375}{T}}\frac{e^{x}x^{4}}{{\left(e^{x}-1\right)}^{2}}dx+\\ 9.4405\times 10^{-6}T+6.9752\times 10^{-6}T^{2}\\ +9.3676\times 10^{-11}T^{4} \end{array}$	9.4405 × 10^−6^	6.9752 × 10^−6^	9.3676 × 10^−11^	375

**Table 4:** Assessed results obtained by extended Einstein model and two-state model.

Phase	
fcc	*Θ_E_* = 320 K
	$\begin{array}{l} G_{Ein}=-8649.2+\frac{3}{2}R\Theta_{E}-3RT\ln\frac{\exp(\Theta_{E}/T)}{\exp(\Theta_{E}/T)-1}\\ -8\times 10^{-4}T^{2}-1.1003\times 10^{-6}T^{3}-1.8512\times 10^{-15}T^{4} \end{array}$
bcc	*Θ_E_* = 330 K
	$\begin{array}{l} G_{Ein}=-8634.5+\frac{3}{2}R\Theta_{E}-3RT\ln\frac{\exp(\Theta_{E}/T)}{\exp(\Theta_{E}/T)-1}\\ -0.0056T^{2}+8.0569\times 10^{-7}T^{3}-2.7097\times 10^{-15}T^{4} \end{array}$
Liquid	*Θ_E_* = 259.9 K
	$\Delta G_{dif}=-27.0883-3.2670T-9.8776T\ln T$
	$\begin{array}{l} G_{p}^{L}=-5132.2+\frac{3}{2}R\Theta_{E}-3RT\ln\frac{\exp\left(\Theta_{E}/T\right)}{\exp\left(\Theta_{E}/T\right)-1}-RT\ln[1+\exp(-\frac{\Delta G_{dif}}{RT})]\\ -0.0026T^{2}+4.8461\times 10^{-7}T^{3}-4.4717\times 10^{-11}T^{4} \end{array}$

**Table 5:** Results of extending SGTE to 0 K.

Phase	
fcc	*Θ_E_* = 275 K
	$\begin{array}{l} G_{Ein}=\frac{3}{2}R\Theta_{E}-3RT\ln\frac{\exp(\Theta_{E}/T)}{\exp(\Theta_{E}/T)-1}-0.0024T^{2}\\ +2.5797\times 10^{-5}T^{3}-5.6509\times 10^{-8}T^{4}-\;8.1070\times 10^{3} \end{array}$
bcc	*Θ_E_* = 291.1 K
	$\begin{array}{l} G_{Ein}=\frac{3}{2}R\Theta_{E}-3RT\ln\frac{\exp(\Theta_{E}/T)}{\exp(\Theta_{E}/T)-1}-0.0179T^{2}\\ +7.0205\times 10^{-5}T^{3}-\;1.0673\times 10^{-7}T^{4}-\;8.2410\times 10^{3} \end{array}$
Liquid	*Θ_E_* = 204 K
	$\begin{array}{l} G_{Ein}=\frac{3}{2}R\Theta_{E}-3RT\ln\frac{\exp(\Theta_{E}/T)}{\exp(\Theta_{E}/T)-1}-0.0187T^{2}\\ +1.1040\times 10^{-4}T^{3}-1.8390\times 10^{-7}T^{4}+\;4.8740\times 10^{3} \end{array}$

## Data Availability

The data presented in this study are available on request from the corresponding author.
